# Pharmacologic interventions for preventing delirium in adult patients after cardiac surgery: Protocol of a systematic review and network meta-analysis

**DOI:** 10.1097/MD.0000000000013881

**Published:** 2018-12-28

**Authors:** Junru Wen, Hai Zeng, Zunjiang Li, Guoxin He, Yueling Jin

**Affiliations:** a Shanghai University of Traditional Chinese Medicine, Shanghai; b Shanghai University of Medicine & Health Sciences, Shanghai; c The Second Clinical College of Guangzhou University of Chinese Medicine, Guangzhou; d The First Clinical College of Guangzhou University of Chinese Medicine, Guangzhou, China.

**Keywords:** cardiac surgery, delirium, meta-analysis, pharmacologic interventions, protocol, systematic review

## Abstract

**Background:**

Delirium is common in adult patients undergoing cardiac surgery and related to a high morbidity and mortality. Although a variety of pharmacologic interventions have been applied in delirium prevention, there is still uncertainty concerning which drug is optimal. Thus, we plan to conduct a systematic review and network meta-analysis (NMA) of published studies to assess the efficacy and safety of pharmacologic interventions for preventing delirium among those patients.

**Methods:**

A systematic literature search will be conducted in Embase, PubMed, and the Cochrane Library. The primary outcome will be the incidence of postoperative delirium. Secondary outcomes will include all-cause mortality and length of hospital or intensive care unit stay. A frequentist NMA will be conducted using Stata version 14.0. The inconsistency between direct and indirect comparisons will be evaluated using a node splitting method. In addition, surface under the cumulative ranking area will be used to evaluate superiority of different treatments.

**Results:**

The findings of our review will be submitted to a peer-reviewed publication.

**Conclusion:**

Our study will generate convincing evidence regarding the effectiveness and safety of different pharmacologic interventions for delirium prevention in cardiac surgery patients.

## Abstract 2

Supplemental Digital Content is available in the text

## 1 Introduction

Delirium, an acute disorder of attention and cognition including both hypoactive and hyperactive forms, often occurs after general anesthesia surgery and medical critical illness.^[1]^ The prevalence of delirium among patients undergoing cardiac surgery is reported to be up to 47%, and it is more prevalent in elderly patients.^[2]^ A cascade of events are initiated by the development of delirium culminating in increased mortality and morbidity, loss of independence, long-term impairment in cognitive function and dementia, longer hospital stay, institutionalization, and high health care costs.^[1–5]^ It has been reported that over $182 billion yearly in 18 countries in Europe and over $164 billion yearly in America are attributable to delirium.^[2,6]^ The etiology of delirium is known to be multifactorial, potential pathophysiologic contributors to delirium include neurotransmitters, proinflammatory markers, physiologic stressors, metabolic disorders, and electrolyte disorders.^[2,7]^

Recently, an increasing number of randomized controlled trials (RCTs) have examined pharmacologic approaches for delirium prevention in the setting of cardiac surgery, involving dexmedetomidine, propofol, midazolam, lorazepam, sevoflurane, morphine, dexamethasone, ketamine, and statins. These drugs with distinct pharmacologic properties have different merits and demerits in clinical practice. In clinical use, clinicians often have difficulty in the choice among multiple alternative drugs, because only a few of available drugs have been compared to one another head-to-head by previous pairwise meta-analyses which typically compare 2 treatment alternatives.

Network meta-analysis (NMA) can compare multiple interventions with respect to the same condition simultaneously, provide both direct and indirect evidence, and allow for evaluation of relative efficacy of treatments which have not been undertaken head-to-head comparisons in RCTs, which is of benefit to clinical practice guidelines.^[8–11]^ Thus, the primary purpose of our study is to compare the efficacy and safety of pharmacologic agents for preventing postoperative delirium following cardiac surgery. For this aim, a comprehensive NMA of RCTs comparing different drugs for prevention of delirium in cardiac surgery patients will be performed.

## 2 Methods

This study will be performed according to the preferred reporting items for systematic review and meta-analysis protocols (PRISMA-P) statement^[12,13]^ and the PRISMA extension statement for NMA.^[8]^ The protocol of our study is registered in the international prospective register of systematic reviews (PROSPERO) (registration no: CRD42018115812).

### 2.1 Inclusion and exclusion criteria for study selection

#### 2.1.1 Types of studies

To be included, studies will be required to investigate different drugs for prevention of delirium in cardiac surgery patients. The study designs will only contain RCTs.

#### 2.1.2 Types of participants

Eligible participants will be those who are adult patients (older than 18 years) undergoing cardiac surgery, regardless of sex, age, region, race, and the type of cardiac surgery.

#### 2.1.3 Types of interventions and controls

We aim to include RCTs investigating the effectiveness and safety on delirium prevention of any of the following nine drugs used perioperatively: dexmedetomidine, propofol, midazolam, lorazepam, sevoflurane, morphine, dexamethasone, ketamine, and statins. RCTs with 2 arms involving these drugs or 1 arm involving these drugs and 1 arm with a placebo control will be included.

#### 2.1.4 Types of outcomes

The primary outcome will be the incidence of postoperative delirium. The evaluation instruments of delirium need be reported (e.g., the Confusion Assessment Method for intensive care unit [ICU]). Secondary outcomes will include all-cause mortality and length of hospital or ICU stay.

#### 2.1.5 Exclusion criteria

Case reports, non-RCTs, quasi-RCTs, observational studies, studies without clinical outcomes of interest, review articles, conference abstracts, comments, animal and in vitro experiments, and duplicated reports will be excluded.

### 2.2 Search strategy

A systematic literature search will be conducted by 2 independent reviewers using Medical Subject Headings (MeSH) and relevant text words. Relevant RCTs will be searched from inception date to November 2018 in following databases: the Cochrane Library database, Embase, and PubMed. The detailed search strategy in PubMed is available in supporting information. Reviewers will resolve any disagreement by discussion. In addition, reference lists from review articles and trials will be manually scrutinized to identify additional related studies.

### 2.3 Data collection

#### 2.3.1 Selection of studies

The records of initial computerized literature search will be imported into EndNote X9 software. According to the prespecified inclusion and exclusion criteria, 2 reviewers will complete the review of potentially qualified articles via screening the titles and abstracts. For remaining studies, each full-text article will be then scrutinized for eligibility by these reviewers. All disagreements in study selection will be settled by consultation. The PRISMA flowchart will be applied to depict the process of study selection.

#### 2.3.2 Data extraction

For each eligible study, using a standardized electronic form, data will be extracted independently and in duplicate by 2 reviewers. Any discrepancy in data extraction among reviewers will be settled through consensus discussion. For each eligible trial, the following information will be extracted: the 1st author, location where the study was performed, year of publication, number and mean age of participants, percent of male participants, intervention and control type, the outcomes of interest, assessment methods of postoperative delirium, and duration of the follow-up.

#### 2.3.3 Dealing with missing data

We will contact authors of primary publications to obtain missing information, if there is a paucity of details of the trial results in the eligible literature. Available data will be applied for statistical analysis when adequate information could not be acquired in this way.

### 2.4 Quality assessment of included studies

To assess the methodologic qualities of included studies, 2 independent reviewers will apply the Cochrane Collaboration tool for evaluating the risk of bias, including following 7 quality items: random sequence generation, allocation concealment, blinding of participants and personnel, blinding of outcome assessment, incomplete outcome data, selective reporting, and other bias.^[14]^ Of those, each item will be classified as low, unclear, or high.

### 2.5 Statistical analysis

#### 2.5.1 Pairwise meta-analysis

Standard pairwise meta-analyses will be conducted with Stata (version 14.0, StataCorp, College Station, TX) software. There will be 2 styles of outcome data: dichotomous and continuous outcomes. Summary odds ratios with 95% confidence intervals (CIs) will be calculated for the former. As to the latter, mean differences corresponding 95% CIs will be estimated as well. A *P*-value of <.05 will be considered statistically significant. The heterogeneity among included studies will be calculated by the *I*^2^ statistic quantitatively.^[15]^ If *I*^2^ statistic value is over 50%, which indicates there exists relatively high heterogeneity across included studies, the aggregated estimate of effects will be calculated by a random-effects model. Otherwise, a fixed-effects model will be applied to synthesize data. Additionally, if substantial heterogeneity is inspected among included studies, to explore potential sources of heterogeneity, we will perform subgroup and meta-regression analyses. The funnel plot and Egger regression test will be performed to assess publication bias when over 10 RCTs are available.

#### 2.5.2 Network meta-analysis

Firstly, a graph of network geometry of the intervention network of comparisons among RCTs will be drawn to evaluate if the NMA is feasible. For a graph of network, the edges correspond to head-to-head comparisons between treatments, and the nodes represent treatment strategies^[8]^. RCTs will be excluded when they could not be connected by interventions. Moreover, the inconsistency between direct and indirect comparisons will be evaluated using a node splitting method when a closed triangle or quadratic loop connecting no <3 arms exists.^[16,17]^ In addition, surface under the cumulative ranking area (SUCRA) will be used to evaluate superiority of different treatments.^[18]^ Generally, a larger SUCRA means a more effective intervention. Furthermore, the comparison-adjusted funnel plots will be used to assess publication bias in the NMA.^[19]^ These statistical analyses above will also be performed with Stata version 14.0.

### 2.6 Grading the quality of evidence

To evaluate the quality of evidence, the grading of recommendations assessment, development and evaluation (GRADE)^[20]^ approach will be used. The level of evidence contains 4 grades as follows: very low, low, moderate, and high.

### 2.7 Ethics and dissemination

This is a meta-analytic review based on published studies; therefore, ethical approval is not required. The findings of our NMA will be submitted to a peer-reviewed publication.

## 3 Discussion

Delirium, an acute confusional state, is characterized by decline of memory, inattention, fluctuating awareness, and disorganized thinking.^[21]^ Moreover, delirium is related to adverse clinical outcomes which cause considerable stress to sufferers as well as families.^[22]^ Thus, it makes sense to target prevention of delirium to those patients at high risk. There still remains a paucity of comparisons and assessments with respect to a variety of treatments, though a number of RCTs have evaluated the effectiveness and safety of pharmacologic interventions for preventing delirium in adult patients after cardiac surgery. To assist clinicians with the prevention of delirium, we plan to conduct a detailed NMA regarding this topic, which will synthesize recent evidence gleaned from all available RCTs. To our knowledge, the present study is the 1st NMA review comparing the efficacy and safety of different drugs on delirium prevention among adult patients undergoing cardiac surgery. Importantly, the findings of our study will be potential to assist the evidence users regarding decision making on delirium prevention for those patients in clinical practice. However, several limitations regarding our review should be acknowledged. The assessment methods of postoperative delirium among included RCTs might be varied. The literature search relied on 3 databases is a further limitation of the present NMA. Moreover, only studies published in English will be included.

## Author contributions

**Conceptualization:** Junru Wen, Hai Zeng.

**Data curation:** Junru Wen, Zunjiang Li, Guoxin He.

**Formal analysis:** Junru Wen, Hai Zeng, Zunjiang Li.

**Funding acquisition:** Junru Wen, Hai Zeng, Zunjiang Li, Yueling Jin.

**Investigation:** Junru Wen, Hai Zeng, Zunjiang Li.

**Methodology:** Junru Wen, Hai Zeng, Zunjiang Li, Yueling Jin.

**Project administration:** Guoxin He.

**Resources:** Hai Zeng, Guoxin He.

**Software:** Junru Wen, Hai Zeng, Zunjiang Li, Yueling Jin.

**Supervision:** Hai Zeng, Junru Wen, Yueling Jin.

**Validation:** Junru Wen, Hai Zeng.

**Visualization:** Junru Wen, Hai Zeng, Zunjiang Li.

**Writing – original draft:** Zunjiang Li, Guoxin He, Junru Wen.

**Writing – review & editing:** Junru Wen, Hai Zeng, Yueling Jin.

## Supplementary Material

Supplemental Digital Content
