# Supplementary material for: Pharmacologic interventions for preventing delirium in adult patients after cardiac surgery: Protocol of a systematic review and network meta-analysis
Source: Medicine (Baltimore). 2018 Dec 28;97(52):e13881. doi: 10.1097/MD.0000000000013881 (PMC6314755; doi:10.1097/MD.0000000000013881)
Supplement: Supplemental Digital Content [file medi-97-e13881-s001.doc]

Search strategy in PubMed.

#1.

Delirium [mesh] OR deliri* [mp] OR "acute confusion*" [tiab] OR "acute organic psychosyndrome"[tiab] OR "acute brain syndrome" [tiab] OR "metabolic encephalopathy" [tiab] OR "acute psycho-organic syndrome" [tiab] OR "clouded state" [tiab] OR "clouding of consciousness" [tiab] OR "exogenous psychosis" [tiab] OR "toxic psychosis" [tiab] OR "toxic confusion" [tiab] OR obnubilat* [tiab]

#2.

"thoracic surgery"[mesh] OR "cardiac surgical procedures"[mesh] ((heart*[mp] OR coronary[tiab] OR "coronary artery*"[mp] OR cardio*[mp] OR cardiac[tiab] OR valve*[mp] OR myocardial[tiab]) AND (surg*[mp] OR intervention*[mp] OR procedure*[mp] OR bypass*[mp]))

#3.

dexmedetomidine [tiab] OR propofol [tiab] OR midazolam [tiab] OR lorazepam [tiab] OR sevoflurane [tiab] OR morphine [tiab] OR dexamethasone [tiab] OR ketamine [tiab] OR statins [tiab]

#4.

"randomized controlled trial"[pt] OR "controlled clinical trial"[pt] OR randomized[tiab] OR placebo[tiab] OR "drug therapy"[sh] OR randomly[tiab] OR trial[tiab] OR groups[tiab]

#5. #1 AND #2 AND #3 AND #4
